# Rethinking Steroid-Induced Rosacea: Why Vascular Laser Therapy Deserves an Earlier Role

**DOI:** 10.7759/cureus.113757

**Published:** 2026-07-31

**Authors:** Yasir Radhi, Ali Almamoori, Hayder Alhamami

**Affiliations:** 1 Department of Dermatology, Al-Shaheed Al-Sadr General Hospital, Baghdad, IRQ; 2 Department of Dermatology, Baghdad Teaching Hospital, Baghdad, IRQ; 3 Department of Dermatology, Baghdad College of Medicine, Baghdad, IRQ

**Keywords:** 577-nm yellow laser, facial erythema, steroid-induced rosacea, telangiectasia, vascular laser

## Abstract

Steroid-induced rosacea (SIR) is traditionally managed through withdrawal of the offending corticosteroid, trigger avoidance, barrier-supportive care, and topical or systemic anti-inflammatory therapy. Although these measures remain essential, persistent erythema, flushing, and telangiectasia may continue to dominate after the acute rebound has stabilized.

This editorial argues for earlier vascular assessment and, in selected clinically stabilized patients with a vascular-predominant phenotype, consideration of vascular-directed treatment rather than automatic deferral until prolonged medical-treatment failure. "Earlier" does not mean immediate treatment during an actively inflamed or barrier-compromised phase; it refers to evaluation after the rebound is no longer escalating and superficial vascular features remain prominent. The proposal is device-agnostic at the conceptual level: the FlexSys 577-nm yellow laser (GME German Medical Engineering GmbH, Erlangen, Germany) provides a clinically relevant example, but no vascular device has established universal superiority in SIR. Dermoscopic vascular morphology may assist patient selection, with superficial network-like vessels appearing more responsive than deeper blue venules. Evidence from conventional rosacea and an uncontrolled prospective SIR study provides biological and clinical rationale but does not establish causality, optimal timing, or superiority of any device. The proposal is therefore hypothesis-generating rather than practice-changing. Controlled comparative studies are needed to define patient selection, timing, device matching, safety, patient-reported outcomes, and long-term durability.

## Editorial

Steroid-induced rosacea (SIR) is an increasingly familiar presentation in dermatology clinics, particularly in regions where potent topical corticosteroids are readily available without prescription. In many such settings, exposure is cosmetic rather than medical. Compounded cosmetic skin-lightening mixtures (locally known as khalta in parts of the Middle East) may be applied to the face for months or years to treat melasma or achieve generalized lightening, often without medical supervision. Although the term khalta is region-specific, unsupervised facial corticosteroid misuse is not; comparable rosacea-like eruptions have been documented in Baghdad and northern India [1,2]. Potent corticosteroids have also been identified in products marketed as cosmetics or "natural" preparations, including illegally adulterated products, further complicating recognition and prevention of exposure [3,4]. The resulting dermatosis may include persistent erythema, flushing, telangiectasia, papules, pustules, burning, dryness, and marked rebound after corticosteroid withdrawal. Observational studies have similarly reported a predominance among women and a high frequency of erythema and telangiectasia after prolonged facial corticosteroid use [1,2].

Terminology in this field remains unsettled. A recent article proposed distinguishing true SIR, understood as rosacea unmasked or exacerbated by corticosteroids in predisposed individuals, from steroid-induced rosacea-like dermatitis (SIRD), in which barrier injury and withdrawal-related inflammation predominate [5]. This proposed distinction has not yet been validated but is clinically relevant to treatment timing. The present argument does not support laser treatment during an actively inflamed, barrier-compromised dermatitis; it applies only after clinical stabilization, when persistent erythema, flushing, and superficial telangiectasia dominate. Throughout this editorial, SIR is used in its conventional broad clinical sense unless otherwise specified.

Current phenotype-based guidance for conventional rosacea recognizes vascular laser and light-based modalities as treatment options for persistent erythema and telangiectasia [6]. However, these recommendations do not define the timing of device-based therapy in corticosteroid-induced disease, particularly during or after withdrawal-related rebound. In clinical practice, management of SIR often prioritizes corticosteroid withdrawal, barrier support, and anti-inflammatory therapy, while vascular treatment is deferred until later. The unresolved question is therefore not whether vascular manifestations can be treated, but whether vascular assessment and selective intervention are sometimes delayed by default. This editorial does not propose an evidence-established treatment algorithm; it advances a cautious, hypothesis-generating case for early vascular assessment and, after clinical stabilization, selective consideration of earlier intervention.

A vascular-predominant phenotype treated mainly as inflammation

For the purposes of this editorial, a vascular-predominant phenotype refers to persistent erythema, recurrent flushing, and clinically or dermoscopically visible superficial telangiectasia that remain prominent when papules, pustules, marked edema, and other signs of active inflammatory instability are absent or comparatively limited. This is a pragmatic clinical description rather than a formally validated classification for SIR.

Although inflammation is integral to the development and expression of SIR, many patients present with a prominent superficial vascular phenotype. Naked-eye examination may record diffuse erythema, whereas dermoscopy can reveal discrete network-like telangiectasia and other superficial vessel patterns. These vascular abnormalities are not merely incidental findings at the margins of an inflammatory eruption; in vascular-predominant cases, they are central to the visible phenotype and to the patient's concern. Persistent facial redness and flushing may remain socially and psychologically burdensome even after papules and pustules begin to improve.

This distinction matters because the principal medical treatments used for SIR are directed primarily toward inflammation. Oral tetracyclines and topical anti-inflammatory agents may improve papules, pustules, burning, edema, and inflammatory erythema, but they do not selectively eliminate established ectatic vessels. The phenotype-based approach to conventional rosacea likewise recognizes that different manifestations require different therapeutic targets rather than expecting a single treatment to control every component of disease [7]. Persistent visible vessels after inflammatory improvement may therefore reflect an untreated structural vascular component rather than failure of medical therapy.

Limits of inflammation-centered treatment

Corticosteroid withdrawal is non-negotiable, because no device-based treatment can compensate for continued exposure to the causative agent. Discontinuation may provoke a clinically unstable rebound characterized by worsening erythema, burning, edema, papules, or pustules, particularly after prolonged use of potent preparations [1,2]. Management should therefore be individualized according to the severity of the flare and the degree of barrier impairment. This unstable phase provides an additional rationale for avoiding immediate or aggressive laser treatment while the rebound is still escalating.

Oral antibiotics and topical calcineurin inhibitors have been used to control inflammatory manifestations. Bhat et al. proposed a combination of oral antibiotics and topical tacrolimus as a preferred approach, though within the limits of an uncontrolled observational design [2]. Such evidence is clinically useful but does not establish comparative efficacy or address whether established telangiectasia should be treated earlier. Medical treatment may reduce inflammatory redness as the flare settles, but fixed telangiectasia remains a different therapeutic target. If prominent superficial vessels continue to dominate the patient's appearance, waiting through prolonged courses of medication before offering a modality designed specifically for those vessels may unnecessarily extend visible disease and dissatisfaction.

Why earlier vascular-directed therapy is biologically plausible

Vascular lasers use selective photothermolysis: light is preferentially absorbed by hemoglobin and converted into thermal energy within the targeted vessel. With appropriate wavelength, pulse duration, fluence, spot size, and cooling, vessel injury can be produced while limiting damage to surrounding tissue.

Pulsed dye laser and intense pulsed light are among the best-studied device-based treatments for persistent erythema and telangiectasia in conventional rosacea. A 2024 meta-analysis found clinically meaningful improvement with both modalities, although it did not establish a universally superior device [8]. Importantly, this evidence derives predominantly from conventional rosacea rather than SIR. It provides a relevant biological and clinical framework, but extrapolation to corticosteroid-induced disease should remain cautious until SIR-specific comparative studies are available.

The 577-nm yellow wavelength is particularly suited to superficial red vessels because it lies close to a major oxyhemoglobin absorption peak. This offers a rational means of targeting the superficial vascular patterns encountered in SIR. Dermoscopic characterization of vessel depth, diameter, color, and arrangement may improve patient selection and help explain differential response.

Persistent superficial vasodilation may contribute to visible erythema and flushing after the acute inflammatory flare begins to settle. Reducing the burden of ectatic superficial vessels could therefore improve the vascular component of the phenotype. Any effect on edema, inflammatory activity, or the corticosteroid-withdrawal rebound remains speculative and should not be inferred from the established vascular effects of laser treatment.

Although our clinical experience relates specifically to the FlexSys 577-nm yellow laser (GME German Medical Engineering GmbH, Erlangen, Germany), the conceptual proposal concerns treatment timing and phenotype-directed care rather than endorsement of a particular wavelength or commercial platform. Device selection should depend on vessel morphology and depth, skin phototype, treatment availability, operator experience, and the safety profile of the chosen modality.

Interpreting our prospective study

In our recently published uncontrolled prospective study of 50 patients with SIR, treatment was performed using the FlexSys 577-nm yellow laser while the causative topical corticosteroid was withdrawn [9]. Mean improvement reached 87.0% for erythema, 79.3% for flushing, and 73.9% for telangiectasia. Superficial network-like vessels were the predominant telangiectatic morphology and generally responded more favorably than deeper blue venules, supporting the view that telangiectasia should not be treated as a uniform entity. However, these findings are exploratory: the absence of a comparator arm and the concurrent withdrawal of topical corticosteroids prevent attribution of the observed improvement to laser treatment alone.

The study also included small clinical subgroups, and follow-up was insufficient to establish long-term recurrence. Furthermore, because the study was designed before the proposed distinction between SIR and SIRD, participants were classified using conventional clinical terminology and were not prospectively stratified according to this emerging framework. The findings therefore cannot determine whether treatment response differs between the proposed entities. They also do not establish that the 577-nm laser is superior to pulsed dye laser, intense pulsed light, potassium titanyl phosphate laser, or other vascular devices.

Our argument does not reclassify the 577-nm laser from an adjunctive intervention to an evidence-established first-line therapy. It concerns timing: an adjunctive vascular treatment may reasonably be considered earlier when persistent erythema and superficial telangiectasia dominate after clinical stabilization. No comparative trial has yet demonstrated that early vascular laser intervention produces better outcomes than delayed treatment in SIR. The proposal is therefore pathophysiology-driven and hypothesis-generating, not a claim of established superiority.

"Earlier" does not mean immediate or indiscriminate

An earlier role does not mean that every patient should receive laser therapy at the first consultation. During an intense rebound flare, the skin may be inflamed, edematous, irritable, and barrier-impaired; immediate aggressive treatment may increase discomfort, edema, or the risk of post-inflammatory pigmentary alteration. "Earlier" should therefore be defined by clinical stabilization rather than a predetermined number of days or weeks.

Earlier integration should begin with prompt vascular assessment and may progress to treatment only after acute inflammatory instability is no longer escalating. No validated definition of clinical stabilization currently exists in SIR. The following should therefore be regarded as pragmatic, unvalidated indicators rather than formal eligibility criteria: cessation of new pustule formation, substantial resolution of marked edema, a clear reduction in burning or stinging, and absence of continued worsening of the withdrawal flare. These indicators should be considered collectively and within the overall clinical context. Patients with predominantly papulopustular disease may initially benefit most from anti-inflammatory therapy, whereas those with persistent erythema, fine superficial telangiectasia, and relatively limited active inflammation may be considered for vascular-directed treatment sooner.

Skin phototype and vessel morphology must guide device selection and parameters. Competing epidermal melanin absorption requires conservative settings, adequate cooling, appropriate intervals, and careful endpoint assessment in darker phototypes. Fine, sharply defined, superficial network-like red vessels appear to represent the most favorable morphology for vascular laser response, whereas deeper blue venules may respond less completely or less predictably, potentially because of their greater depth and different optical characteristics [9]. Such deeper or larger vessels may require a different wavelength or treatment strategy. The argument is therefore not for indiscriminate use of one device, but against requiring every patient to exhaust prolonged medical therapy before the vascular component can be considered.

A practical phenotype-directed sequence

A practical phenotype-directed sequence can be summarized in four stages. First, identify and withdraw the causative corticosteroid while initiating gentle cleansing, barrier-supportive moisturization, photoprotection, trigger avoidance, and counseling regarding withdrawal-related rebound. Second, control active inflammatory instability according to the severity of papules, pustules, burning, edema, and other inflammatory manifestations. Third, assess and document the vascular phenotype clinically and dermoscopically; this assessment may occur early and does not imply immediate treatment. Fourth, once clinical stabilization has been achieved, selectively consider vascular laser treatment when superficial erythema and telangiectasia remain prominent. This progression distinguishes the relatively strong rationale for early vascular recognition and assessment from the still-unproven benefit of earlier intervention.

In mixed phenotypes, anti-inflammatory and vascular-directed treatments may be used sequentially or in combination, with the balance determined by the dominant clinical features. Serial dermoscopic assessment, preferably using standardized images of the same facial areas, may help document changes in vessel density, caliber, and morphology, identify residual vessels that are less apparent to the naked eye, and assess the durability of vascular clearance or subsequent recurrence.

This integrated model does not replace medical management. The coexistence of nonvascular manifestations further emphasizes that vascular laser therapy should be integrated with, rather than substituted for, treatments directed at the inflammatory, follicular, and barrier-related components of SIR.

Real-world implementation also depends on access. Device availability, operator expertise, treatment cost, and the possibility that vascular procedures may not be reimbursed can limit early intervention, particularly in resource-constrained settings. These barriers do not invalidate a phenotype-directed approach, but they should be acknowledged when translating the proposal into routine practice and when designing comparative studies.

What future studies must establish

The priority is a controlled comparison between conventional management alone and conventional management combined with early vascular laser therapy. Studies should standardize corticosteroid withdrawal, concomitant medication, laser parameters, treatment intervals, and follow-up. Stratification should include skin phototype, corticosteroid potency and duration, baseline inflammatory activity, the proposed SIR-SIRD distinction, and dermoscopic vascular morphology. Outcomes should include objective erythema measures, vascular scores, symptom improvement, validated patient-reported quality-of-life and satisfaction measures, time to satisfactory clearance, recurrence, and adverse events.

Direct comparisons between vascular devices are also needed. The aim should not be to declare one wavelength universally superior, but to determine which vascular morphology and skin phenotype are best matched to each modality.

Conclusion

Vascular laser therapy should not be promoted as universal first-line monotherapy for SIR. Corticosteroid withdrawal, barrier restoration, and control of active inflammation remain essential. However, relegating vascular treatment to a rescue intervention after prolonged failure of medical therapy is also difficult to justify when persistent erythema and telangiectasia dominate the phenotype.

The most defensible position lies between these extremes: early recognition and dermoscopic assessment of the vascular phenotype, followed, only after inflammatory stabilization, by selective consideration of vascular intervention. Earlier assessment is justified by the need to identify the dominant phenotype, whereas the clinical benefit of earlier treatment remains a testable hypothesis requiring controlled comparative evidence. The question is not whether the vascular component can be treated, but whether it is sometimes deferred by default rather than addressed according to phenotype, clinical stability, device suitability, and patient priorities.
